# Incorporation of desmocollin‐2 into the plasma membrane requires *N*‐glycosylation at multiple sites

**DOI:** 10.1002/2211-5463.12631

**Published:** 2019-04-03

**Authors:** Andreas Brodehl, Caroline Stanasiuk, Dario Anselmetti, Jan Gummert, Hendrik Milting

**Affiliations:** ^1^ Erich and Hanna Klessmann Institute for Cardiovascular Research & Development (EHKI) Heart and Diabetes Center NRW University Hospital of the Ruhr‐University Bochum Oeynhausen Germany; ^2^ Faculty of Physics, Experimental Biophysics and Applied Nanoscience Bielefeld Institute for Nanoscience (BINAS) Bielefeld University Germany

**Keywords:** arrhythmogenic (right ventricular) cardiomyopathy, desmocollin‐2, desmosomes, *N*‐glycosylation, *O*‐mannosylation, vesicle transport

## Abstract

Desmocollin‐2 (DSC2) is a desmosomal protein of the cadherin family. Desmosomes are multiprotein complexes, which are involved in cell adhesion of cardiomyocytes and of keratinocytes. The molecular structure of the complete extracellular domain (ECD) of DSC2 was recently described, revealing three disulfide bridges, four *N*‐glycosylation sites, and four *O*‐mannosylation sites. However, the functional relevance of these post‐translational modifications for the protein trafficking of DSC2 to the plasma membrane is still unknown. Here, we generated a set of DSC2 mutants, in which we systematically exchanged all *N*‐glycosylation sites, *O*‐mannosylation sites, and disulfide bridges within the ECD and investigated the resulting subcellular localization by confocal laser scanning microscopy. Of note, all single and double *N*‐glycosylation‐ deficient mutants were efficiently incorporated into the plasma membrane, indicating that the absence of these glycosylation sites has a minor effect on the protein trafficking of DSC2. However, the exchange of multiple *N*‐glycosylation sites resulted in intracellular accumulation. Colocalization analysis using cell compartment trackers revealed that *N*‐glycosylation‐ deficient DSC2 mutants were retained within the Golgi apparatus. In contrast, elimination of the four *O*‐mannosylation sites or the disulfide bridges in the ECD has no obvious effect on the intracellular protein processing of DSC2. These experiments underscore the importance of *N*‐glycosylation at multiple sites of DSC2 for efficient intracellular transport to the plasma membrane.

**Abbreviations**
ACM: arrhythmogenic (right ventricular) cardiomyopathy
DSC2: desmocollin‐2
DSG2: desmoglein‐2
ECD: extracellular domain
MAF: minor allele frequency
PG: plakoglobin
PKP2: plakophilin‐2
PTM: post‐translational modification

Regulation of cell adhesion is highly relevant for cells exposed to mechanical stress such as cardiomyocytes during heart contraction or keratinocytes during skin stretching. Desmosomes are specialized multiprotein complexes, which mediate cell–cell adhesion 1. Mutations in genes, encoding desmosomal proteins, cause different genetic diseases of the skin and/or the heart such as striate palmoplantar keratoderma (MIM #148700) 2, Naxos disease (MIM #601214) 3, Carvajal syndrome (MIM #605676) 4, or arrhythmogenic (right ventricular) cardiomyopathy (ACM; MIM #609040) 5, 6, respectively. ACM is clinically characterized by right or biventricular dilation and severe ventricular arrhythmias leading to heart failure or even sudden cardiac death 7. Histologically, ACM is caused by fibrofatty replacement of the myocardial tissue 8. About 50% of the ACM patients carry one or more mutations in genes encoding structural proteins of the cardiac desmosomes 5, 9.

The structural proteins forming the desmosomes belong to three protein families. Desmocollin‐2 (DSC2) and desmoglein‐2 (DSG2) are members of the cadherin superfamily and connect the cardiomyocytes 10. The desmosomal cadherins are type I transmembrane proteins and consist of an intracellular C‐terminal domain, a transmembrane domain, and five N‐terminal extracellular domains (ECD1‐ECD5) 10, 11. The protein–protein interactions between the desmosomal cadherins are Ca^2+^‐dependent and are mediated by their first ECDs 10. The intracellular cytoplasmic domains are connected to plakophilin‐2 (PKP2) and plakoglobin (PG), which are members of the Armadillo family 12. PKP2 and PG connect the cadherins to the cytolinker protein desmoplakin, which mediates the interaction with the intermediate filament system of the cell 13.

Recently, Harrison *et al*. 10 determined the molecular structure of the ECDs of different desmosomal cadherins including DSC2 by X‐ray diffraction analysis. This study revealed three different kinds of post‐translational protein modifications (PTMs) within the ECDs of DSC2. Four *N*‐glycosylation sites at p.N166, p.N392, p.N546, and p.N629, four *O*‐mannosylation sites at p.T338, p.T340, p.T558, and p.T560, and three disulfide bridges at p.C471‐C559, p.C585‐C671, and p.C669‐C677 are present in the ECDs of DSC2 (Fig. 1A) 10. *O*‐mannosylations are rare PTMs, which were initially discovered in yeast 14. However, recently several reports identified *O*‐mannosylations also in different members of the human cadherin family 15, 16, 17, 18, 19.

**Figure 1:**
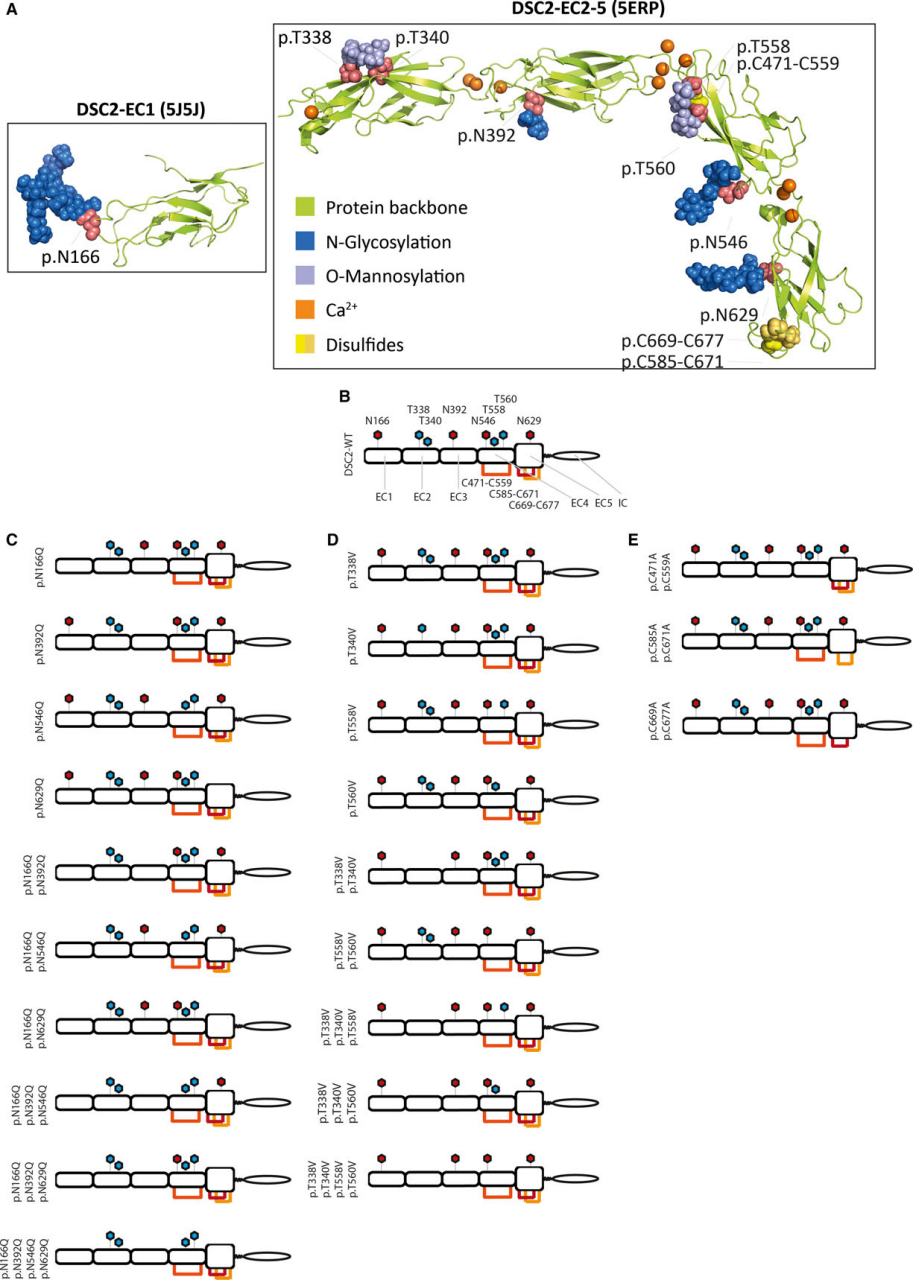
Structural overview about the ECD of DSC2. (A) Molecular structures of the EC1 (5J5J) and EC2‐5 fragments of DSC2 (5ERP, https://www.rcsb.org) 10. The sugar residues of the *N*‐glycosylation sites are shown in dark blue, and the mannose residues of the *O*‐mannosylation sites are shown in light blue. The protein backbone is colored in green, Ca^2+^ ions are shown in orange, and cysteine residues forming disulfide bridges are labeled yellow. (B‐E) Schematic overviews about the generated DSC2 constructs. *N*‐glycosylation sites are shown in red, and *O*‐mannosylation sites are labeled in blue. Disulfide bridges are indicated by yellow and red lines. (B) DSC2 wild‐type construct. (C) *N*‐glycosylation‐ deficient DSC2 constructs. (D) *O*‐mannosylation‐ deficient constructs. (E) Disulfide bridge‐deficient DSC2 constructs.

Nevertheless, the functional impact of these PTMs on the localization of DSC2 is widely unknown. Therefore, we generated a set of different single and multiple DSC2 mutants, which were deficient for single or multiple PTMs (Fig. 1B‐E). We investigated the localization of wild‐type and mutant DSC2 at the plasma membrane by confocal microscopy. These experiments revealed that multiple *N*‐glycosylation‐ deficient DSC2 mutants were intracellularly retained within the Golgi apparatus and were less efficiently incorporated into the plasma membrane. However, genetic deletion of the disulfide bridges or *O*‐mannosylation sites had no obvious effect on the DSC2 localization. Our study highlights the relevance of multiple *N*‐glycosylations for an efficient trafficking of DSC2 to the plasma membrane.

## Materials and methods

### Plasmid generation

The cDNA of DSC2 (NM_024422.4) was fused via polymerase chain reaction (PCR) at the 5′‐end with a *Xho*I and at the 3′‐end with an *Age*I restriction site (Fig. S1) and was afterward cloned into pEYFP‐N1 (Clontech, Mountain View, CA, USA). Missense mutations were inserted using the QuikChange Lightning Site‐Directed Mutagenesis Kit (Agilent Technologies, Santa Clara, CA, USA) using appropriate primers (Table S1). The protein coding regions of all generated plasmids were verified by Sanger sequencing (Macrogen, Amsterdam, the Netherlands). Information about the generated plasmids is summarized in Table S1.

### Cell culture

HT1080 cells were cultured under standard conditions (37 °C, 5% CO_2_, humidified incubator) in Dulbecco's modified Eagle's medium (DMEM, 4.5 g·L^−1^ glucose, supplemented with penicillin and streptomycin and 10% FBS) 20. HL‐1 cardiomyocytes were cultured as previously described.

### Cell transfection and nucleofection

HT1080 cells were transfected using Lipofectamine 3000 (Life Technologies, Carlsbad, CA, USA) according to the manufacturer's instructions. For imaging, cells were cultured in Lab‐Tek II Chambers (Thermo Fisher Scientific, Waltham, MA, USA). Forty eight hours after transfection, the cells were washed twice with PBS and were fixed with Roth‐Histofix 4% (Carl Roth, Karlsruhe, Germany) for 10 min at room temperature (RT).

Briefly, HL‐1 cardiomyocytes were incubated with trypsin/EDTA for 4 min at 37 °C, washed with PBS, and centrifuged for 5 min at 200 ***g***. Afterward, HL‐1 cells were transfected via nucleofection using the 4D‐Nucleofector in combination with the Primary P3 Transfection Kit (Lonza, Cologne, Germany). Thirty micro gram of plasmid DNA was used for nucleofection. After nucleofection, the cells were incubated for 10 min at 37 °C and were cultured afterward in Claycomb medium under standard conditions in Lab‐Tek II Chambers. Forty eight hours after nucleofection, the cells were fixed, stained, and analyzed by confocal microscopy.

### Immunocytochemistry

After fixation with 4% PFA, the cells were permeabilized using 0.1% Triton X‐100 (5 min, RT) and were incubated with rabbit anti‐DSG2 antibodies (Abcam, Cambridge, UK, #ab150372, 1 : 100) for 1 h at RT. Afterward, the cells were washed several times with PBS and were incubated with secondary anti‐rabbit IgG antibodies conjugated with Alexa 647 (Abcam, #ab150075, 1 : 100). After several additional washing steps with PBS, the cells were embedded in Vectashield Antifade Mounting Medium (Vector Laboratories, Burlingame, CA, USA) and were analyzed using confocal microscopy. F‐actin was costained with phalloidin conjugated to Texas Red according to the manufacturer's instructions (Thermo Fisher Scientific, #T7471).

### Confocal microscopy

HT1080 cells were grown in Lab‐Tek II Chamber Slides (Thermo Fisher Scientific) and were directly used for microscopy. Twenty four hours after transfection, the cells were washed with PBS and then fixed. The TCS SP8 system (Leica Microsystems, Wetzlar, Germany), equipped with a HC PL API C52 (63×/1.30) glycerin objective, HyD hybrid detectors, and Application Suite X software, was used for confocal microscopy. EYFP was excited at 488 nm, and the emission was detected between 493 and 560 nm. Alexa 647 was excited at 638 nm, and the emission was detected between 643 and 775 nm. Texas Red was excited at 552 nm, and the emission was detected between 557 and 750 nm. In multichannel experiments, the fluorescence dyes were sequentially imaged.

### Colocalization and cell compartment analysis

Cell compartment trackers were used according to the manufacturer's instructions (Life Technologies). TagRFP was excited at 552 nm, and the emission was detected in the range between 557 and 781 nm. EYFP was excited at 488 nm, and the emission was detected between 493 and 547 nm. The different channels were sequentially imaged. Colocalization analysis was performed using Leica Application Suite X software (Leica Microsystems).

### Western blot analysis

Transfected cells were harvested 48 h after transfection, were washed twice with PBS, and were immediately frozen in liquid nitrogen. The cells were lysed using RIPA buffer (10 mm Tris/HCl, 1 mm EDTA, 0,5 mm EGTA, 1% Triton X‐100, 0,1% SDS, 0,1% sodium deoxycholate, 140 mm NaCl, 1 mm PMSF) supplemented with proteinase inhibitors (Sigma‐Aldrich, St. Louis, MO, USA). Three cycles of freezing in liquid nitrogen and thawing were used for cell lysis. Protein concentrations were determined using the Pierce 660 nm Protein Assay (Thermo Fisher Scientific) according to the manufacturer's instructions. Mini‐PROTEAN 4–20% TGX gels (Bio‐Rad, Hercules, CA, USA) were used for sodium dodecyl sulfate–polyacrylamide electrophoresis (SDS/PAGE). Proteins were blotted using the Trans‐Blot Turbo Transfer System (Bio‐Rad). Homogenous protein transfer on the membranes was verified by Ponceau S staining. After blocking with 5% fat dried milk in Tris‐buffered saline supplemented with 0.1% Tween‐20 (TBST) for 1 h at RT, the membranes were incubated with primary antibodies at 4 °C overnight. Anti‐EYFP (Chromotek, Planegg‐Martinsried #PABG1, 1 : 1000) and anti‐GAPDH (Abcam, #AB9485, 1 : 1000) antibodies were used in combination with secondary antibodies conjugated with horseradish peroxidase (GE Healthcare Life Sciences, Pittsburgh, PN, USA #NA934VS, 1 : 1000). The WesternBright Quantum Detection Kit (Advanstar, Santa Monica, CA, USA) was used in combination with the MultiImage Light Cabinet (Alpha Innotech Corporation, San Leandro, CA, USA) for visualization of the luminescence signals.

### *In silico* analysis

We used the molecular structures of the ECD fragments of DSC2 (5J5J and 5ERP, https://www.rcsb.org/), which were recently reported by Harrison *et al*. 10 for *in silico* analysis using PyMOL 2.1.1 (Schrodinger, Cambridge, MA, USA).

### Statistical analysis

Each transfection experiment was performed in triplicate or more, and about twenty transfected cells were analyzed per experiment. Western blot analysis was repeated twice of two independent transfection experiments revealing comparable results. Data were presented as mean ± standard deviation (SD). Nonparametric Kruskal–Wallis test was performed using graphpad prism v5.00 (GraphPad Software, San Diego, CA, USA). *P*‐values < 0.05 were considered as significant.

## Results

**Figure 2:**
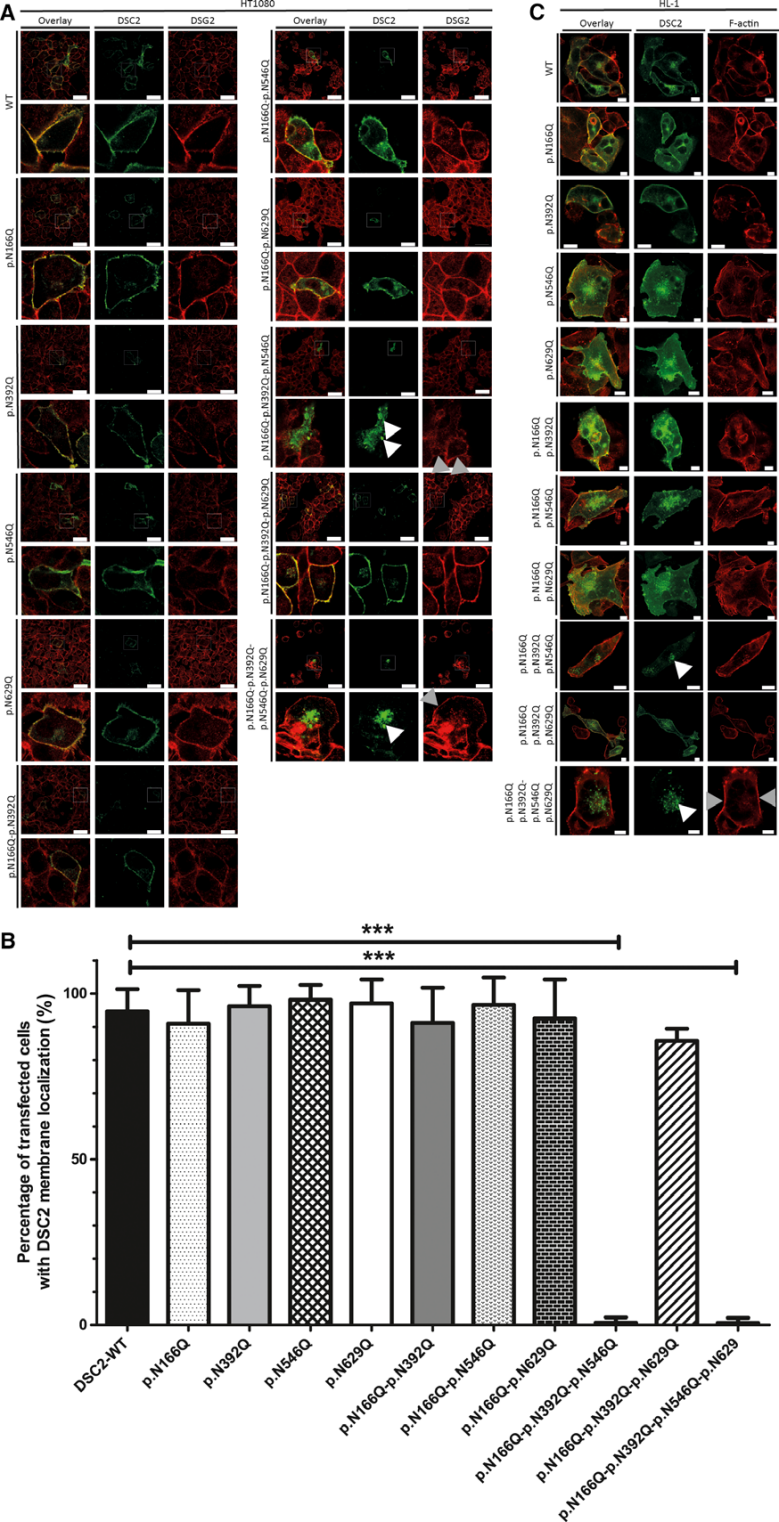
Cellular localization of *N*‐glycosylation‐ deficient DSC2 mutants. (A) Representative fluorescence images and corresponding magnifications of transfected HT1080 cells expressing wild‐type DSC2‐eYFP or *N*‐glycosylation‐ deficient mutants are shown (green). Endogenous DSG2 was stained using anti‐DSG2 antibodies and is shown in red. Scale bars represent 50 μm. (B) Quantitative analysis of transfected cells with DSC2 membrane localization. Nonparametric Kruskal–Wallis test was used for statistical analysis. ***P < 0.001; *n* = 6. Error bars indicate mean ± SD. (C) Representative fluorescence images of transfected HL‐1 cells expressing wild‐type DSC2‐eYFP or *N*‐glycosylation‐ deficient mutants are shown (green). Endogenous F‐actin was stained using phalloidin conjugated to Texas Red. Scale bars represent 10 μm. White arrows indicate localization within the Golgi apparatus, and gray arrowheads indicate plasma membrane localization.

The impact of the PTMs on protein localization of DSC2 is currently unknown. Therefore, we started our analysis by systematically subsidizing the four asparagine residues at p.N166, p.N392, p.N546, and p.N629 against glutamine residues to generate single *N*‐glycosylation‐ deficient DSC2 mutants (Fig. 2). Analysis of transiently transfected HT1080 and HL‐1 cells using confocal microscopy revealed that all DSC2 single mutants deficient for one specific *N*‐glycosylation site were colocalized together with the endogenous DSG2 at the plasma membrane comparable to the wild‐type form (Fig. 2A‐C), indicating that the absence of one *N*‐glycosylation can be compensated. Therefore, we generated systematically multiple *N*‐glycosylation‐ deficient mutants. Whereas the three different double mutants with two deficient *N*‐glycosylation sites were correspondingly localized at the plasma membrane (Fig. 2A–C), the simultaneous deletion of three or four *N*‐glycosylation sites caused an intracellular accumulation of DSC2 in vesicular structures (Fig. 2A–C) in nearly all transfected cells (Fig. 2A–C). These experiments indicate that multiple *N*‐glycosylation sites are necessary for an efficient protein transport to the plasma membrane. Western blot analysis demonstrated a significant different molecular mass of the *N*‐glycosylation‐ deficient mutant in comparison with the wild‐type form (Fig. S3).

**Figure 3:**
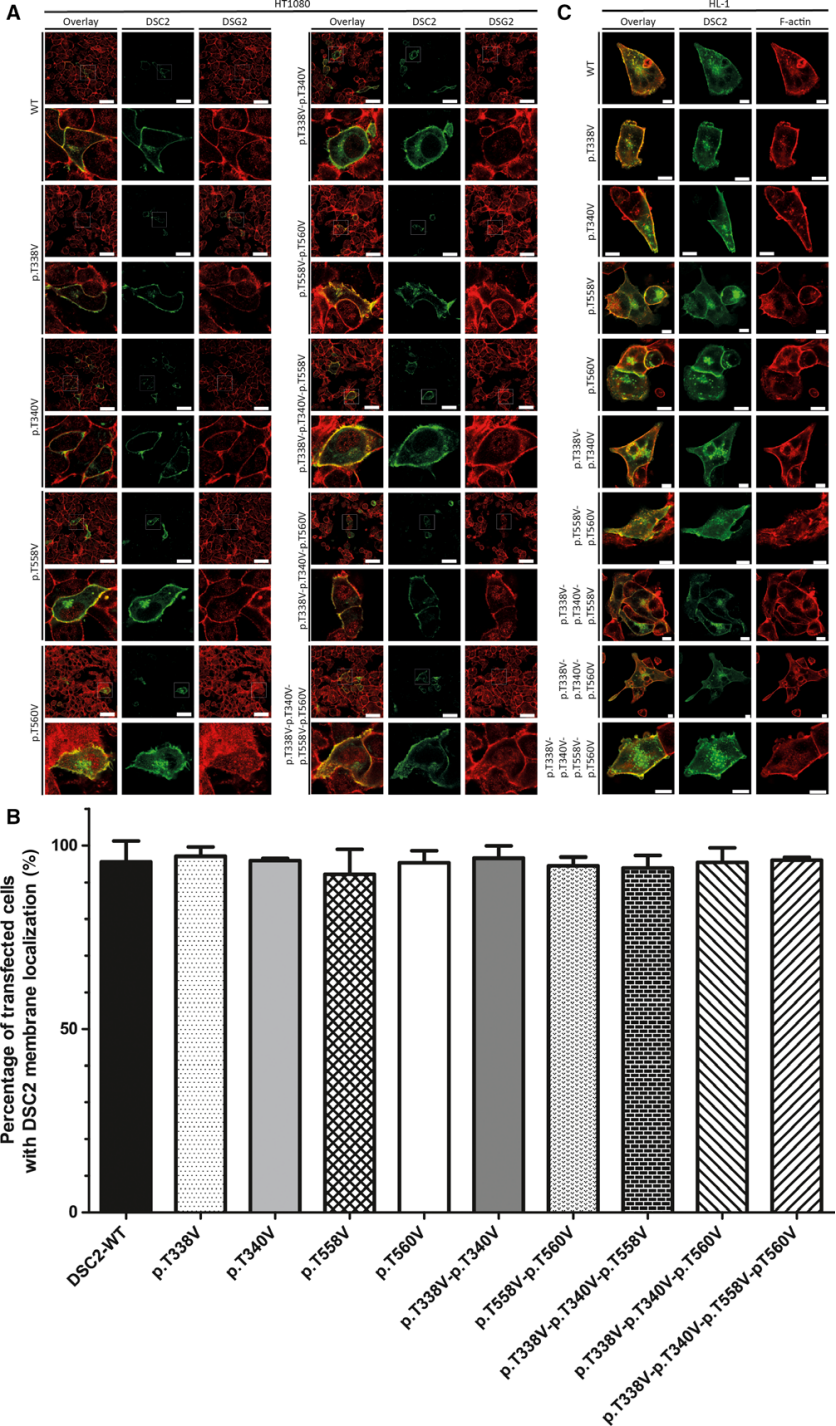
Cellular localization of *O*‐mannosylation‐ deficient DSC2 mutants. (A) Representative fluorescence images of transfected HT1080 cells expressing wild‐type DSC2‐eYFP or *O*‐mannosylation‐ deficient mutants are shown (green). Endogenous desmoglein‐2 is shown in red. Scale bars represent 50 μm. (B) Quantification of transfected HT1080 cells with DSC2 membrane localization revealed no significant differences between wild‐type and disulfide bridge‐deficient DSC2 mutants. Nonparametric Kruskal–Wallis test was used for statistical analysis. *n* = 3. Error bars indicate mean ± SD. (C) Representative fluorescence images of transfected HL‐1 cells expressing wild‐type DSC2‐eYFP (green) and disulfide bridge‐deficient mutants are shown. Endogenous F‐actin is labeled with phalloidin conjugated with Texas Red (red). Scale bars represent 10 μm.

In the next steps, we deleted sequentially the four *O*‐mannosylation sites of DSC2 by changing threonine to valine to investigate the impact of these PTMs on DSC2 trafficking. The four *O*‐mannosylation‐ deficient mutants (p.T338V, p.T340V, p.T558V, and p.T560V) were mainly incorporated into the plasma membrane comparable to the wild‐type DSC2 (Fig. 3). Interestingly, also the double, triple, and quadruple *O*‐mannosylation‐ deficient mutants were mainly colocalized with endogenous DSG2 at the plasma membrane in both cell lines (Fig. 3). Of note, even deletion of all four *O*‐mannosylation sites caused in contrast to the quadruple *N*‐glycosylation‐ deficient mutants no intracellular accumulation in the majority of transfected cells (Fig. 3). These results indicate that the four *O*‐mannosylation sites of DSC2 are not mandatory for an efficient transport to the plasma membrane.

**Figure 4:**
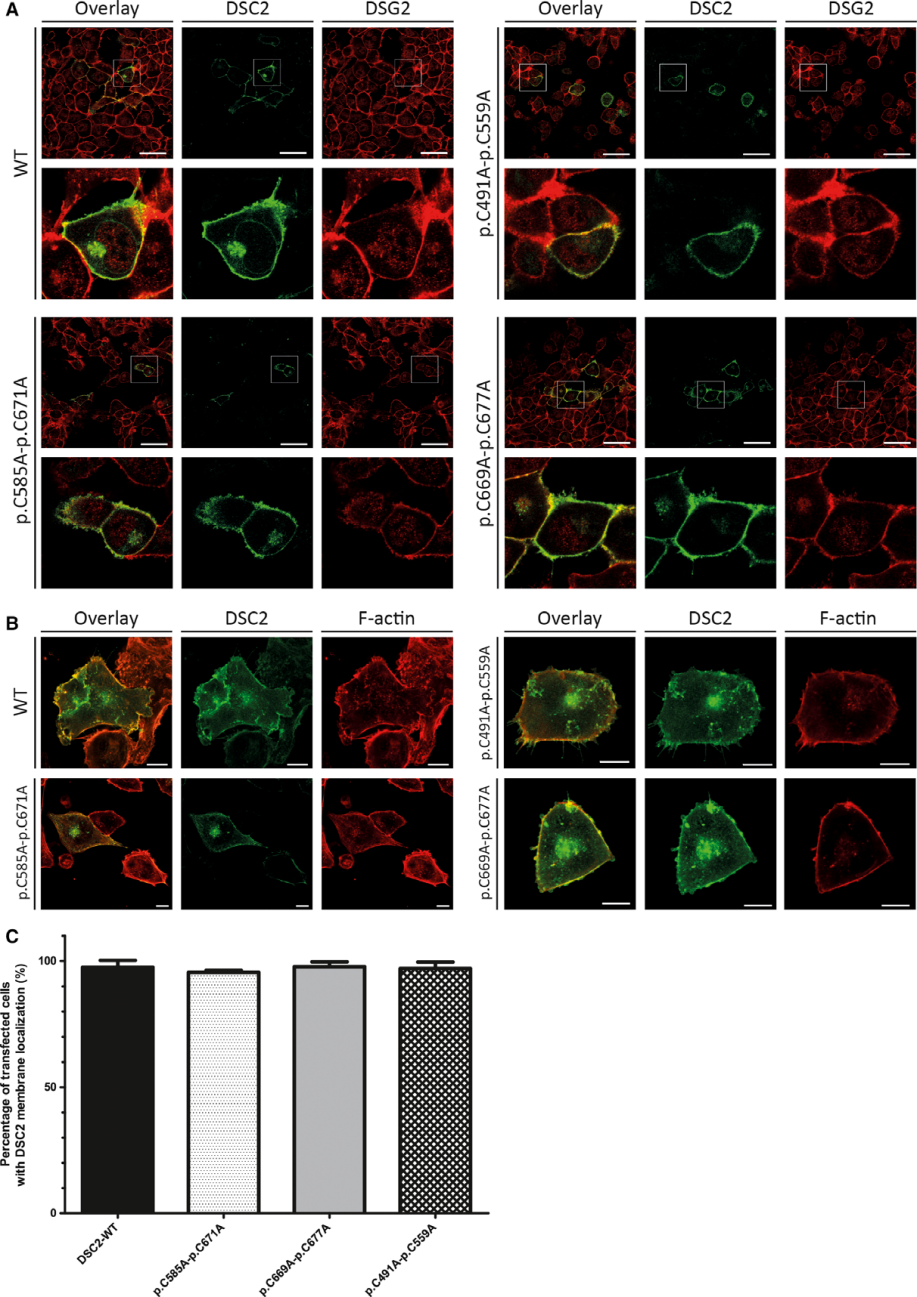
Cellular localization of disulfide bridge‐deficient DSC2 mutants. (A) Representative fluorescence images of transfected HT1080 cells expressing wild‐type DSC2‐eYFP or disulfide bridge‐deficient mutants are shown (green). Endogenous desmoglein‐2 labeled with antibodies is shown in red. Scale bars represent 50 μm. (B) Quantification of transfected HT1080 cells with DSC2 membrane localization revealed no significant differences between wild‐type and disulfide bridge‐deficient DSC2 mutants. Nonparametric Kruskal–Wallis test was used for statistical analysis. *n* = 3. Error bars indicate mean ± SD. (C) Representative fluorescence images of transfected HL‐1 cells expressing wild‐type DSC2‐eYFP or disulfide bridge‐deficient mutants are shown (green). Endogenous F‐actin is labeled with phalloidin conjugated with Texas Red (red). Scale bars represent 10 μm.

Desmocollin‐2 carries in addition also three disulfide bridges (p.C471‐C559, p.C585‐C671, and p.C669‐C677) localized in the ECD4 and ECD5. To evaluate the impact of these PTMs, we generated double DSC2 mutants exchanging both cysteine residues against alanine residues. However, these three disulfide bridge‐deficient DSC2 mutants were not obviously differently localized in comparison with the wild‐type form in transfected HT1080 and HL‐1 cells (Fig. 4A–C) indicating no or only a minor influence on the protein processing of DSC2.

**Figure 5:**
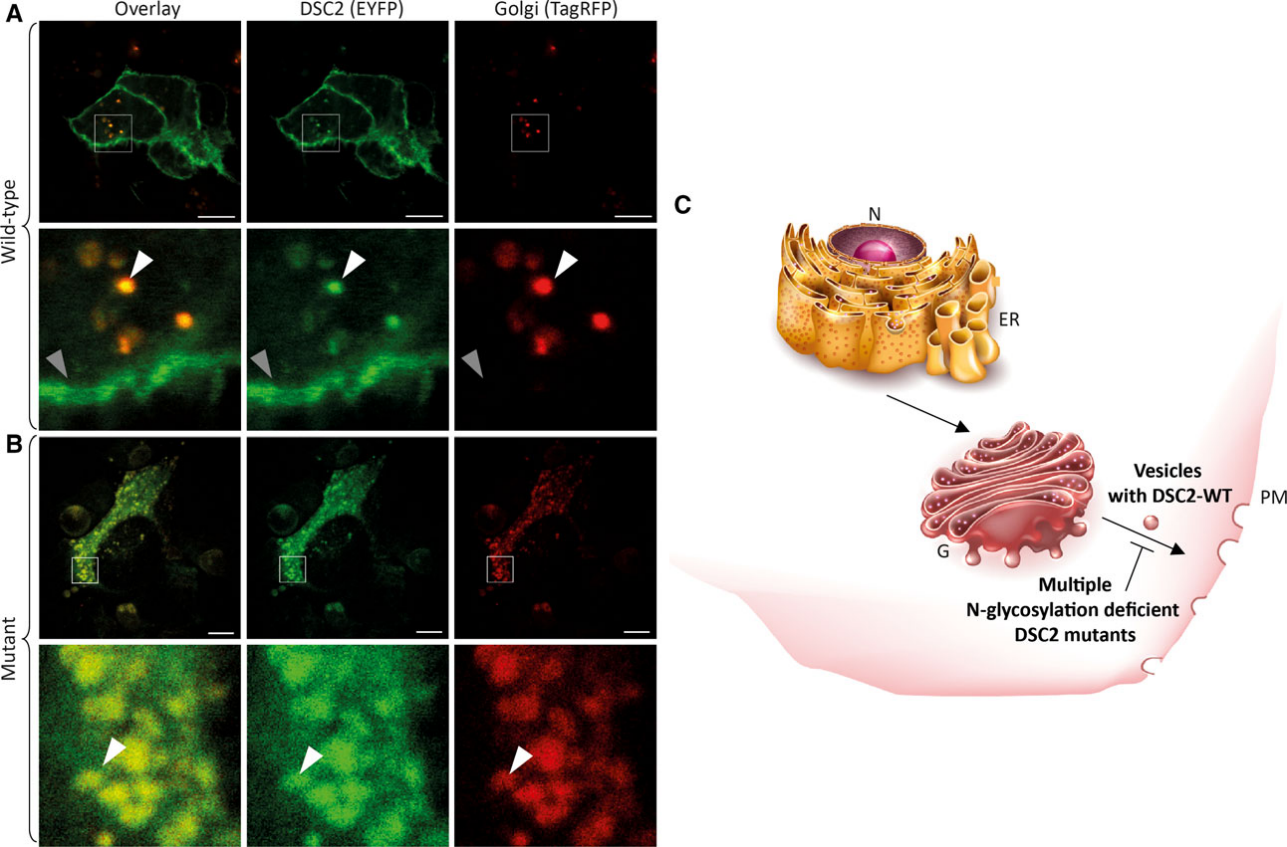
Colocalization analysis of compartment trackers and the *N*‐glycosylation‐ deficient DSC2 mutants. HT1080 cells were transfected with (A) wild‐type and (B) mutant DSC2‐p.N166Q‐p.N392Q‐p.N546Q‐p.N629Q‐p.T338V‐p.T340‐p.T558V‐p.T560V constructs. Cell organelles were stained using compartment trackers. Colocalization of DSC2‐eYFP and the compartment trackers were evaluated using Leica Application Suite X software. Pearson's correlation of DSC2 mutant: 0.83; overlap coefficient: 0.88; colocalization rate: 85.4%. Scale bars represent 10 μm. (C) Schematic overview. ER, endoplasmic reticulum; G, Golgi apparatus; N, nucleus; PM, plasma membrane. (Images for ER and G are licenced from shutterstock.de)

As predicted, the combined deletions of *N*‐glycosylation and *O*‐mannosylation sites caused the same DSC2 accumulation within the intracellular vesicular structures (Fig. S2). To investigate in which cell compartment the DSC2 mutants were retained, we used different cell compartment trackers and investigated the colocalization of wild‐type and mutant DSC2 with these markers. These experiments revealed that the mutant DSC2 molecules were mainly retained within the Golgi apparatus (Fig. 5).

## Discussion

Desmosomes are cell–cell junctions, which received medical attention because mutations in genes encoding desmosomal structural proteins cause severe diseases of the heart 5, 6, 21 and/or of the skin 22. The main function of the desmosomes is to provide cell–cell adhesion, which is important for tissue integrity 23. Especially, cells exposed to nanomechanical stress such as cardiomyocytes during the contraction cycle or epithelial cells during skin stretching express therefore desmosomal proteins. While the structural composition and structure of the desmosomes have been investigated since many years, the cellular regulation and dynamic assembly of desmosomes are poorly understood.

Human‐induced pluripotent stem cells (hiPSCs) can be differentiated into cardiomyocytes 24. However, because genome‐editing approaches of hiPSC‐derived cardiomyocytes to introduce missense variants are still challenging, we used HT1080 cells and in addition the cardiac cell line HL‐1 25, 26. The transfection efficiency of HT1080 cells was in our experiments between 5% and 10% and of HL‐1 cells about 2%. HT1080 cells express endogenous DSG2 but do not express endogenously DSC2 26 and are therefore an appropriate model to investigate DSC2 mutants without interferences of the endogenous protein. In addition, we verified our findings using the cardiac HL‐1 cell line.

Recently, Harrison *et al*. 10 determined the molecular structure of the complete ECDs of DSC2 and DSG2 by X‐ray diffraction. Four different *N*‐glycosylation sites, four *O*‐mannosylation sites, and three disulfide bridges were found in the ECD of DSC2 10. However, the relevance of these PTMs for the protein processing of the desmosomal cadherins is currently unknown. Therefore, we addressed in this study the question which of these PTMs are mandatory for an efficient incorporation of DSC2 into the plasma membrane. To our surprise, genetic deletion of single isolated *N*‐glycosylations, *O*‐mannosylations sites, or disulfide bridges had no or only a minor effect on the protein transport of DSC2 to the plasma membrane, indicating that deficiency of single PTMs can be compensated. Remarkably, the *O*‐mannosylations and disulfide bridges do not play a prominent role for the intracellular transport of DSC2. However, we cannot exclude that the adhesive properties of DSC2 were altered by these PTMs.

Several *DSC2* missense 27, nonsense 28, frameshift 5, 29, or splice site mutations 21, 30 were associated with ACM in humans (ARVC Database, http://www.arvcdatabase.info; 31). However, the clinical relevance of *DSC2* missense mutations is under controversial debate because in most cases the evidence for their pathogenicity is still missing. To the best of our knowledge, no knock‐in mouse model mimicking human ACM is available for DSC2 mutations. Only a transgenic mouse model with a cardiac‐specific overexpression of human wild‐type DSC2 was reported 32. These transgenic mice developed biventricular cardiomyopathy associated with severe fibrosis, inflammatory remodeling, and calcification 32.

Interestingly, there is only one report describing a putative ACM‐associated *DSC2* missense mutation affecting a PTM site 33. Barahona‐Dussault *et al*. 33 identified the *DSC2* missense variant (p.T340A) affecting the second *O*‐mannosylation site within the second ECD in a patient with ACM. However, this patient carries also a second pathogenic frameshift mutation in *PKP2* (p.V837fsX930) complicating the clinical and genetic interpretation of *DSC2*‐p.T340A. In addition, this variant and a second variant at the same position (p.T340N) are also present in control individuals (minor allele frequency, MAF; p.T340A = 0.00003612; p.T340N = 0.00005288; Genome Aggregation Database (gnomAD), http://gnomad.broadinstitute.org/, June 2018) 34. Therefore, the pathogenic impact of *DSC2*‐p.T340A is questionable. This is in good agreement with our functional data demonstrating no obvious effect for single *O*‐mannosylation‐deficient mutants. Interestingly, the gnomAD contains also rare variants of some further PTM sites affecting the disulfide bridges or the *O*‐mannosylation s in the fourth and fifth ECDs (MAF: p.C471Y and p.C471S = 4.067 × 10^−6^; p.C559Y = 4.082 × 10^−6^; p.C671Y = 8.15 × 10^−6^; p.T560M = 1.633 × 10^−5^; and p.T560A = 8.159 × 10^−6^). In spite of this, we could not find obvious effects on the localization of the single mutants missing the corresponding PTMs.

To investigate whether the PTMs might have a cooperative impact for the trafficking and localization of DSC2, we generated a set of multiple *N*‐glycosylation and *O*‐mannosylation‐ deficient mutants. These experiments revealed that obviously multiple *N*‐glycosylation‐ deficient mutants but not multiple *O*‐mannosylation‐ deficient mutants were retained in the Golgi apparatus demonstrating the cooperative relevance of these PTMs for DSC2 processing. However, we have not investigated the cellular half‐life of different DSC2 mutants in comparison with the wild‐type DSC2, which is beyond the scope of this manuscript. Whereas *N*‐glycosylations are found in many human proteins, there are currently only some human proteins known, which carry *O*‐mannosylations at specific serine or threonine residues. However, in the last decade it was shown that especially members of the cadherin family carry *O*‐mannosylations 15, 17, 18, 19. Remarkably, Larsen *et al*. 15 recently demonstrated using a CRISPR/Cas9 genetic dissection approach that the *TMTC1‐4* gene family encodes O‐mannosyltransferases mediating the modification of the cadherin superfamily including DSC2.

In summary, we have investigated in this study the impact of all different known PTMs (*N*‐glycosylation, *O*‐mannosylation, and disulfide bridges) within the ECDs of DSC2 on protein trafficking and incorporation into the plasma membrane. Our study demonstrates that absence of single PTMs can be tolerated, whereas multiple deletions of *N*‐glycosylations lead to an intracellular retention within the Golgi apparatus highlighting the relevance of these PTMs for protein processing of DSC2 (Fig. 5C).

## Conflict of interest

The authors declare no conflict of interest.

## Author contributions

AB and HM conceived and supervised the study. AB designed the experiments. AB and CS performed the experiments. AB analyzed the data. AB wrote the manuscript. AB, JG, DA, and HM acquired the funding. DA, JG, and HM made manuscript revisions.

## Supporting information


**Fig. S1.** Overview about the cloning strategy. DSC2a cDNA was amplified by PCR fusing a *Xho*I and *Age*I restriction site. Afterward the DSC2 cDNA was inserted into pEYFP‐N1 (Clontech). Sequential site directed mutagenesis was used to insert the mutations. The protein coding regions of all plasmids were sequenced (Macrogen).

**Fig. S2**. (A‐D) Schematic overviews about the generated multiple PTM deficient DSC2 cDNA constructs. *N*‐glycosylation sites are shown in red and *O*‐mannosylation sites are labeled in blue. Representative fluorescence images and corresponding magnifications of transfected HT1080 cells expressing wild‐type DSC2‐eYFP (green) and *N*‐glycosylation deficient mutants are shown. (A) DSC2‐p.N166Q‐p.T338V‐p.T340V‐p.N392Q‐p.N546Q (B) DSC2–p.N166Q‐p.N392Q‐p.N546Q‐p.T558V‐p.T560V; (C) DSC2‐p.N166Q‐p.N392Q‐p.N546Q‐p.N629Q‐p.T338V‐p.T340V (D) p.N166Q‐p.N392Q‐p.N546Q‐p.N629Q‐p.T338V‐p.T340V‐p.T558V‐p.T560V. Scale bars represent 10 μm.

**Fig. S3.** (A‐F) Electropherograms demonstrating the replacement of modified amino acids. (G) Western blot analysis revealed the expression of DSC2‐EYFP fusion constructs in transfected cells. Of note, the molecular mass of the *N*‐glycosylation deficient construct is significant smaller in comparison to the wild‐type form. GAPDH was used as a loading control.

**Table S1.** Overview about the generated plasmids and used oligonucleotides (5′‐3′).

## References

[1] Patel DM and Green KJ (2014) Desmosomes in the heart: a review of clinical and mechanistic analyses. Cell Commun Adhes 21, 109–128. 24754498 10.3109/15419061.2014.906533

[2] Nomura T , Mizuno O , Miyauchi T , Suzuki S , Shinkuma S , Hata H , Fujita Y , Akiyama M and Shimizu H (2015) Striate palmoplantar keratoderma: report of a novel DSG1 mutation and atypical clinical manifestations. J Dermatol Sci 80, 223–225. 26493105 10.1016/j.jdermsci.2015.10.004

[3] McKoy G , Protonotarios N , Crosby A , Tsatsopoulou A , Anastasakis A , Coonar A , Norman M , Baboonian C , Jeffery S and McKenna WJ (2000) Identification of a deletion in plakoglobin in arrhythmogenic right ventricular cardiomyopathy with palmoplantar keratoderma and woolly hair (Naxos disease). Lancet 355, 2119–2124. 10902626 10.1016/S0140-6736(00)02379-5

[4] Norgett EE , Hatsell SJ , Carvajal‐Huerta L , Cabezas JC , Common J , Purkis PE , Whittock N , Leigh IM , Stevens HP and Kelsell DP (2000) Recessive mutation in desmoplakin disrupts desmoplakin‐intermediate filament interactions and causes dilated cardiomyopathy, woolly hair and keratoderma. Hum Mol Genet 9, 2761–2766. 11063735 10.1093/hmg/9.18.2761

[5] Klauke B , Kossmann S , Gaertner A , Brand K , Stork I , Brodehl A , Dieding M , Walhorn V , Anselmetti D , Gerdes D *et al* (2010) *De novo* desmin‐mutation N116S is associated with arrhythmogenic right ventricular cardiomyopathy. Hum Mol Genet 19, 4595–4607. 20829228 10.1093/hmg/ddq387

[6] Gerull B , Heuser A , Wichter T , Paul M , Basson CT , McDermott DA , Lerman BB , Markowitz SM , Ellinor PT , MacRae CA *et al* (2004) Mutations in the desmosomal protein plakophilin‐2 are common in arrhythmogenic right ventricular cardiomyopathy. Nat Genet 36, 1162–1164. 15489853 10.1038/ng1461

[7] Vimalanathan AK , Ehler E and Gehmlich K (2018) Genetics of and pathogenic mechanisms in arrhythmogenic right ventricular cardiomyopathy. Biophys Rev 10, 973–982. 29995277 10.1007/s12551-018-0437-0 PMC6082309

[8] El Demellawy D , Nasr A and Alowami S (2009) An updated review on the clinicopathologic aspects of arrhythmogenic right ventricular cardiomyopathy. Am J Forensic Med Pathol 30, 78–83. 19237863 10.1097/PAF.0b013e318187379e

[9] Xu T , Yang Z , Vatta M , Rampazzo A , Beffagna G , Pilichou K , Scherer SE , Saffitz J , Kravitz J , Zareba W *et al* (2010) Compound and digenic heterozygosity contributes to arrhythmogenic right ventricular cardiomyopathy. J Am Coll Cardiol 55, 587–597. 20152563 10.1016/j.jacc.2009.11.020 PMC2852685

[10] Harrison OJ , Brasch J , Lasso G , Katsamba PS , Ahlsen G , Honig B and Shapiro L (2016) Structural basis of adhesive binding by desmocollins and desmogleins. Proc Natl Acad Sci USA 113, 7160–7165. 27298358 10.1073/pnas.1606272113 PMC4932976

[11] Koch PJ and Franke WW (1994) Desmosomal cadherins: another growing multigene family of adhesion molecules. Curr Opin Cell Biol 6, 682–687. 7833048 10.1016/0955-0674(94)90094-9

[12] Chen X , Bonne S , Hatzfeld M , van Roy F and Green KJ (2002) Protein binding and functional characterization of plakophilin 2. Evidence for its diverse roles in desmosomes and beta ‐catenin signaling. J Biol Chem 277, 10512–10522. 11790773 10.1074/jbc.M108765200

[13] Choi HJ , Park‐Snyder S , Pascoe LT , Green KJ and Weis WI (2002) Structures of two intermediate filament‐binding fragments of desmoplakin reveal a unique repeat motif structure. Nat Struct Biol 9, 612–620. 12101406 10.1038/nsb818

[14] Sentandreu R and Northcote DH (1968) The structure of a glycopeptide isolated from the yeast cell wall. Biochem J 109, 419–432. 5685868 10.1042/bj1090419 PMC1186836

[15] Larsen ISB , Narimatsu Y , Joshi HJ , Siukstaite L , Harrison OJ , Brasch J , Goodman KM , Hansen L , Shapiro L , Honig B *et al* (2017) Discovery of an O‐mannosylation pathway selectively serving cadherins and protocadherins. Proc Natl Acad Sci USA 114, 11163–11168. 28973932 10.1073/pnas.1708319114 PMC5651762

[16] Larsen ISB , Narimatsu Y , Joshi HJ , Yang Z , Harrison OJ , Brasch J , Shapiro L , Honig B , Vakhrushev SY , Clausen H *et al* (2017) Mammalian O‐mannosylation of cadherins and plexins is independent of protein O‐mannosyltransferases 1 and 2. J Biol Chem 292, 11586–11598. 28512129 10.1074/jbc.M117.794487 PMC5500819

[17] Carvalho S , Oliveira T , Bartels MF , Miyoshi E , Pierce M , Taniguchi N , Carneiro F , Seruca R , Reis CA , Strahl S *et al* (2016) O‐mannosylation and N‐glycosylation: two coordinated mechanisms regulating the tumour suppressor functions of E‐cadherin in cancer. Oncotarget 7, 65231–65246. 27533452 10.18632/oncotarget.11245 PMC5323151

[18] Baenziger JU (2013) O‐mannosylation of cadherins. Proc Natl Acad Sci USA 110, 20858–20859. 24344310 10.1073/pnas.1321827111 PMC3876236

[19] Lommel M , Winterhalter PR , Willer T , Dahlhoff M , Schneider MR , Bartels MF , Renner‐Müller I , Ruppert T , Wolf E and Strahl S (2013) Protein O‐mannosylation is crucial for E‐cadherin‐mediated cell adhesion. Proc Natl Acad Sci USA 110, 21024–21029. 24297939 10.1073/pnas.1316753110 PMC3876218

[20] Preusser‐Kunze A , Mariappan M , Schmidt B , Gande SL , Mutenda K , Wenzel D , von Figura K and Dierks T (2005) Molecular characterization of the human Calpha‐formylglycine‐generating enzyme. J Biol Chem 280, 14900–14910. 15657036 10.1074/jbc.M413383200

[21] Heuser A , Plovie ER , Ellinor PT , Grossmann KS , Shin JT , Wichter T , Basson CT , Lerman BB , Sasse‐Klaassen S , Thierfelder L *et al* (2006) Mutant desmocollin‐2 causes arrhythmogenic right ventricular cardiomyopathy. Am J Hum Genet 79, 1081–1088. 17186466 10.1086/509044 PMC1698714

[22] Kato M , Shimizu A , Yokoyama Y , Kaira K , Shimomura Y , Ishida‐Yamamoto A , Kamei K , Tokunaga F and Ishikawa O (2015) An autosomal recessive mutation of DSG4 causes monilethrix through the ER stress response. J Invest Dermatol 135, 1253–1260. 25615553 10.1038/jid.2015.12

[23] Johnson JL , Najor NA and Green KJ (2014) Desmosomes: regulators of cellular signaling and adhesion in epidermal health and disease. Cold Spring Harb Perspect Med 4, a015297. 25368015 10.1101/cshperspect.a015297 PMC4208714

[24] Barbuti A , Benzoni P , Campostrini G and Dell'Era P (2016) Human derived cardiomyocytes: a decade of knowledge after the discovery of induced pluripotent stem cells. Dev Dyn 245, 1145–1158. 27599668 10.1002/dvdy.24455

[25] Gaertner A , Klauke B , Stork I , Niehaus K , Niemann G , Gummert J and Milting H (2012) *In vitro* functional analyses of arrhythmogenic right ventricular cardiomyopathy‐associated desmoglein‐2‐missense variations. PLoS One 7, e47097. 23071725 10.1371/journal.pone.0047097 PMC3468437

[26] Chitaev NA and Troyanovsky SM (1997) Direct Ca2 + ‐dependent heterophilic interaction between desmosomal cadherins, desmoglein and desmocollin, contributes to cell‐cell adhesion. J Cell Biol 138, 193–201. 9214392 10.1083/jcb.138.1.193 PMC2139935

[27] Gehmlich K , Syrris P , Peskett E , Evans A , Ehler E , Asimaki A , Anastasakis A , Tsatsopoulou A , Vouliotis AI , Stefanadis C *et al* (2011) Mechanistic insights into arrhythmogenic right ventricular cardiomyopathy caused by desmocollin‐2 mutations. Cardiovasc Res 90, 77–87. 21062920 10.1093/cvr/cvq353 PMC3058729

[28] Gerull B , Kirchner F , Chong JX , Tagoe J , Chandrasekharan K , Strohm O , Waggoner D , Ober C and Duff HJ (2013) Homozygous founder mutation in desmocollin‐2 (DSC2) causes arrhythmogenic cardiomyopathy in the Hutterite population. Circ Cardiovasc Genet 6, 327–336. 23863954 10.1161/CIRCGENETICS.113.000097

[29] Gehmlich K , Lambiase PD , Asimaki A , Ciaccio EJ , Ehler E , Syrris P , Saffitz JE and McKenna WJ (2011) A novel desmocollin‐2 mutation reveals insights into the molecular link between desmosomes and gap junctions. Heart Rhythm 8, 711–718. 21220045 10.1016/j.hrthm.2011.01.010 PMC3085091

[30] Groeneweg JA , Ummels A , Mulder M , Bikker H , van der Smagt JJ , van Mil AM , Homfray T , Post JG , Elvan A , van der Heijden JF *et al* (2014) Functional assessment of potential splice site variants in arrhythmogenic right ventricular dysplasia/cardiomyopathy. Heart Rhythm 11, 2010–2017. 25087486 10.1016/j.hrthm.2014.07.041

[31] van der Zwaag PA , Jongbloed JD , van den Berg MP , van der Smagt JJ , Jongbloed R , Bikker H , Hofstra RM and van Tintelen JP (2009) A genetic variants database for arrhythmogenic right ventricular dysplasia/cardiomyopathy. Hum Mutat 30, 1278–1283. 19569224 10.1002/humu.21064

[32] Brodehl A , Belke DD , Garnett L , Martens K , Abdelfatah N , Rodriguez M , Diao C , Chen YX , Gordon PM , Nygren A *et al* (2017) Transgenic mice overexpressing desmocollin‐2 (DSC2) develop cardiomyopathy associated with myocardial inflammation and fibrotic remodeling. PLoS One 12, e0174019. 28339476 10.1371/journal.pone.0174019 PMC5365111

[33] Barahona‐Dussault C , Benito B , Campuzano O , Iglesias A , Leung TL , Robb L , Talajic M and Brugada R (2010) Role of genetic testing in arrhythmogenic right ventricular cardiomyopathy/dysplasia. Clin Genet 77, 37–48. 19863551 10.1111/j.1399-0004.2009.01282.x

[34] Lek M , Karczewski KJ , Minikel EV , Samocha KE , Banks E , Fennell T , O'Donnell‐Luria AH , Ware JS , Hill AJ , Cummings BB *et al* (2016) Analysis of protein‐coding genetic variation in 60,706 humans. Nature 536, 285–291. 27535533 10.1038/nature19057 PMC5018207

