# Supplementary figures and images for: Incorporation of desmocollin‐2 into the plasma membrane requires N‐glycosylation at multiple sites
Source: FEBS Open Bio. 2019 Apr 3;9(5):996–1007. doi: 10.1002/2211-5463.12631 (PMC6487837; doi:10.1002/2211-5463.12631)

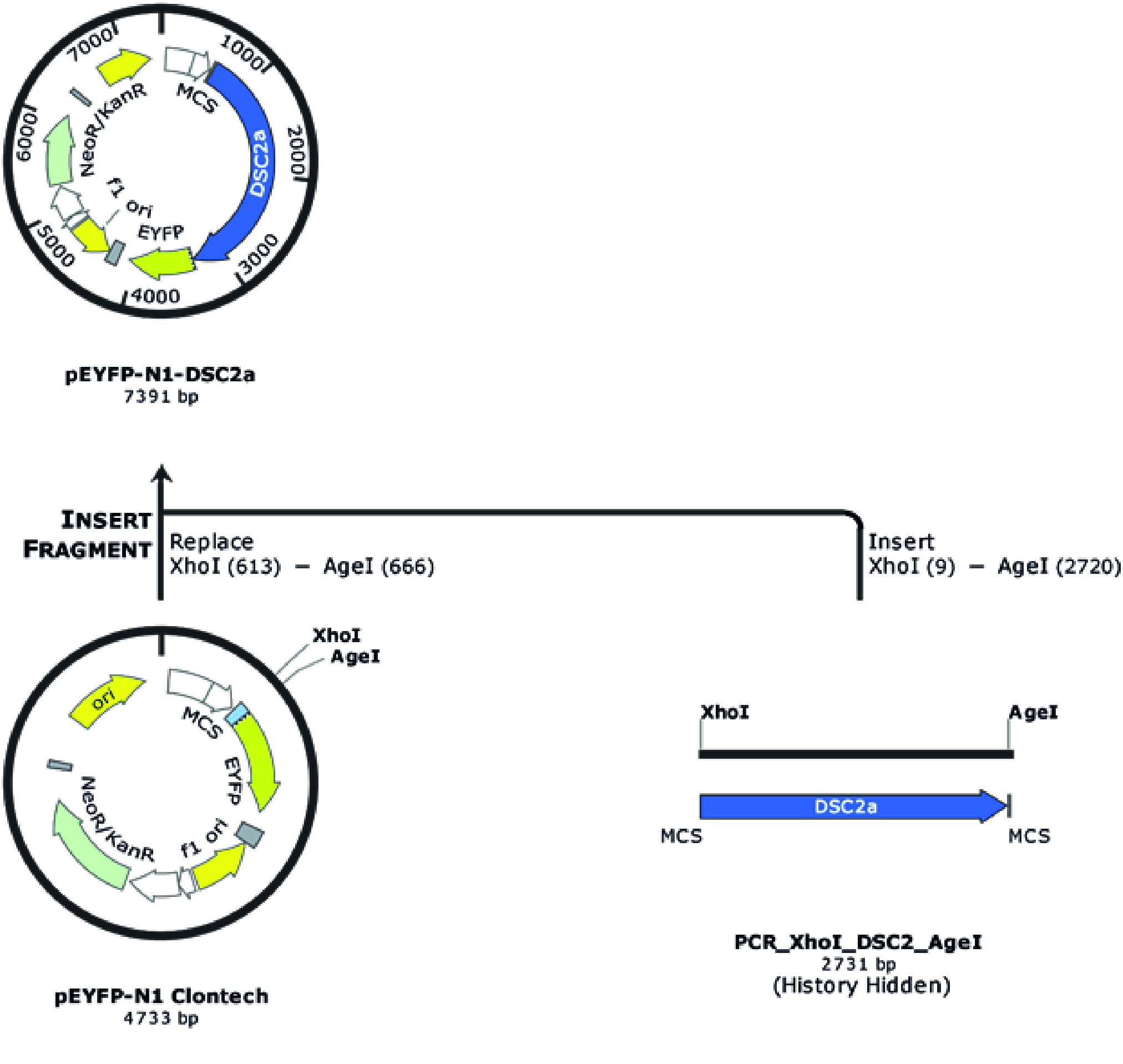

Supplement: Supplementary file 1 — Fig. S1. Overview about the cloning strategy. DSC2a cDNA was amplified by PCR fusing a XhoI and AgeI restriction site. Afterward the DSC2 cDNA was inserted into pEYFP‐N1 (Clontech). Sequential site directed mutagenesis was used to insert the mutations. The protein coding regions of all plasmids were sequenced (Macrogen). [file FEB4-9-996-s001.tif]

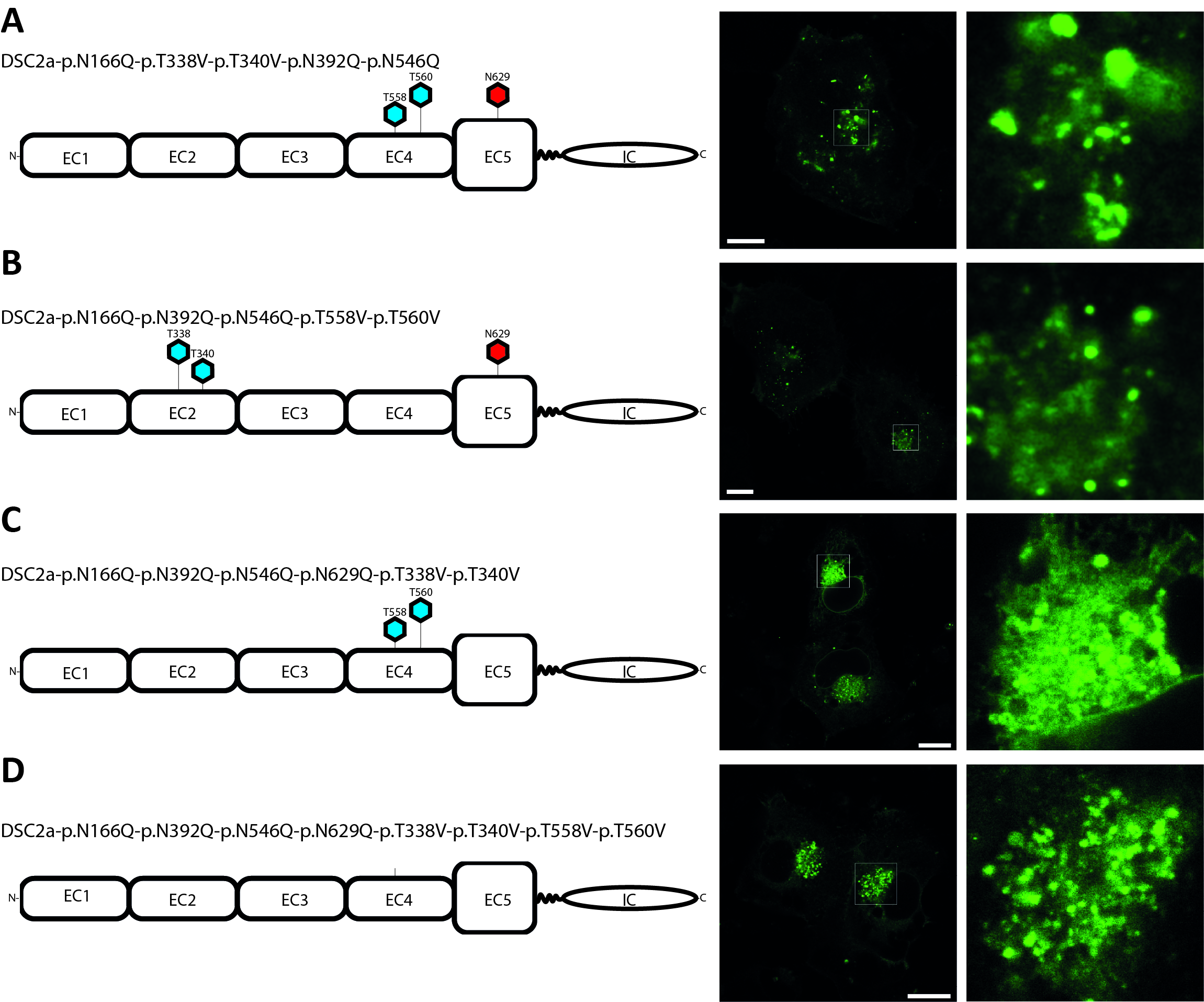

Supplement: Supplementary file 2 — Fig. S2. (A‐D) Schematic overviews about the generated multiple PTM deficient DSC2 cDNA constructs. N‐glycosylation sites are shown in red and O‐mannosylation sites are labeled in blue. Representative fluorescence images and corresponding magnifications of transfected HT1080 cells expressing wild‐type DSC2‐eYFP (green) and N‐glycosylation deficient mutants are shown. (A) DSC2‐p.N166Q‐p.T338V‐p.T340V‐p.N392Q‐p.N546Q (B) DSC2–p.N166Q‐p.N392Q‐p.N546Q‐p.T558V‐p.T560V; (C) DSC2‐p.N166Q‐p.N392Q‐p.N546Q‐p.N629Q‐p.T338V‐p.T340V (D) p.N166Q‐p.N392Q‐p.N546Q‐p.N629Q‐p.T338V‐p.T340V‐p.T558V‐p.T560V. Scale bars represent 10 μm. [file FEB4-9-996-s002.tif]

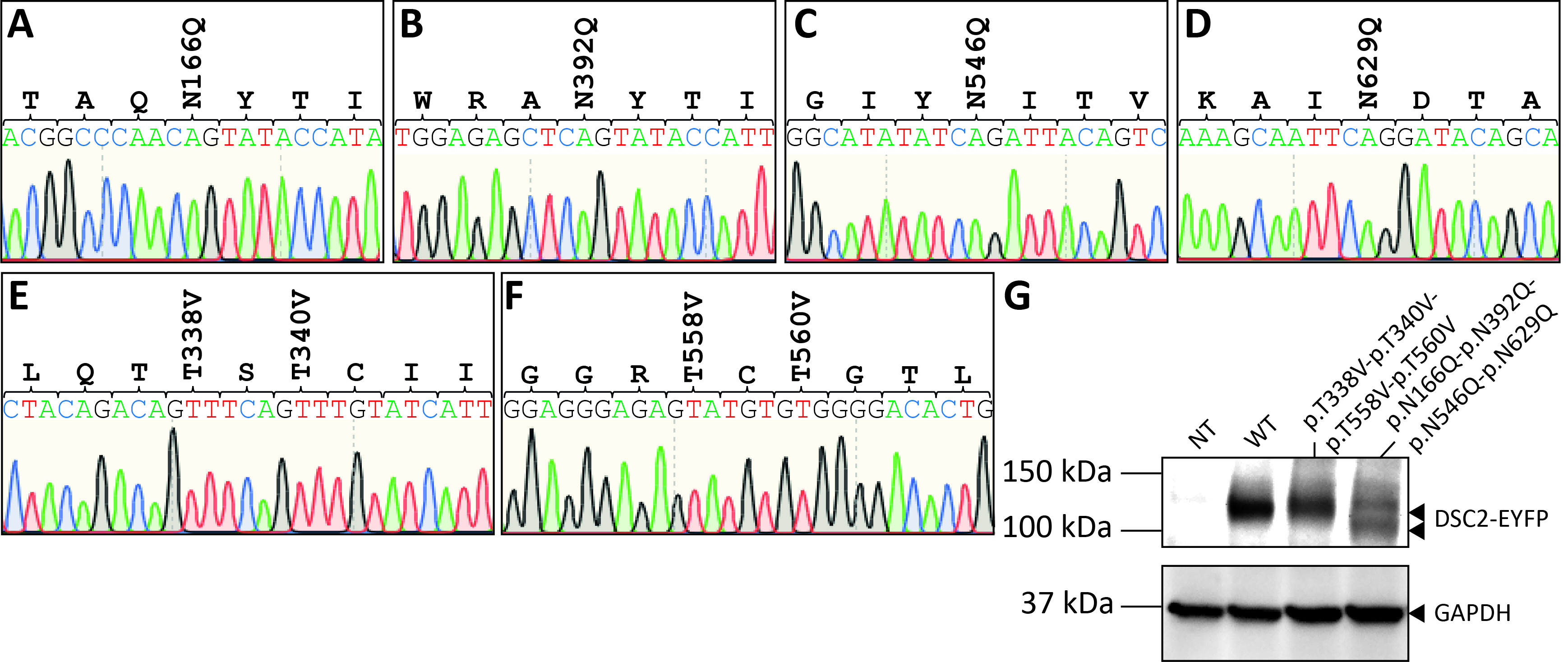

Supplement: Supplementary file 3 — Fig. S3. (A‐F) Electropherograms demonstrating the replacement of modified amino acids. (G) Western blot analysis revealed the expression of DSC2‐EYFP fusion constructs in transfected cells. Of note, the molecular mass of the N‐glycosylation deficient construct is significant smaller in comparison to the wild‐type form. GAPDH was used as a loading control. [file FEB4-9-996-s003.tif]
