# Supplementary material for: Incorporation of desmocollin‐2 into the plasma membrane requires N‐glycosylation at multiple sites
Source: FEBS Open Bio. 2019 Apr 3;9(5):996–1007. doi: 10.1002/2211-5463.12631 (PMC6487837; doi:10.1002/2211-5463.12631)
Supplement: Supplementary file 4 — Table S1. Overview about the generated plasmids and used oligonucleotides (5′‐3′). [file FEB4-9-996-s004.docx]

**Table S1.** Overview about the generated plasmids and used oligonucleotides (5’-3’)

[1**]** GCGGCCGCCTCGAGATGGAGGCAGCCCGCC; [2] GGATCCACCGGTCCTCTCTTCATGCATGCTTCTGCTAGTGTCC; [3] GGTTCAATCTGACACGGCCCAACAGTATACCATATACTATTCCATAAG; [4] CTTATGGAATAGTATATGGTATACTGTTGGGCCGTGTCAGATTGAACC; [5] ATGGTCAGTATTTTGGTCTACAGACAGTTTCAACTTGTATCATTAACATTGATG; [6] CATCAATGTTAATGATACAAGTTGAAACTGTCTGTAGACCAAAATACTGACCAT; [7] CATTTACATCATCAATGTTAATGATACAAACTGAAGTTGTCTGTAGACCAAAATACTGAC; [8] GTCAGTATTTTGGTCTACAGACAACTTCAGTTTGTATCATTAACATTGATGATGTAAATG; [9] GTGAATACTGCTAACTGGAGAGCTCAGTATACCATTTTAAAGGGCAATGAA; [10] TTCATTGCCCTTTAAAATGGTATACTGAGCTCTCCAGTTAGCAGTATTCAC; [11] GATGCAAGGACTGTAATCTGATATATGCCATTTTTGATGGTCTCTGCC; [12] GGCAGAGACCATCAAAAATGGCATATATCAGATTACAGTCCTTGCATC; [13] CCAGTGTCCCCGTACATACTCTCCCTCCTTGGTCTG; [14] CAGACCAAGGAGGGAGAGTATGTACGGGGACACTGG; [15] CCAGTGTCCCCACACATGTTCTCCCTCCTTGGTC; [16] GACCAAGGAGGGAGAACATGTGTGGGGACACTGG; [17] ACGTGCTGCTGTATCCTGAATTGCTTTCAGTCTCCACATTCTC; [18] GAGAATGTGGAGACTGAAAGCAATTCAGGATACAGCAGCACGT; [19] CATCAATGTTAATGATACAAACTGAAACTGTCTGTAGACCAAAATACTGACCAT; [20] ATGGTCAGTATTTTGGTCTACAGACAGTTTCAGTTTGTATCATTAACATTGATG; [21] CCAGTGTCCCCACACATACTCTCCCTCCTTGGTCTG; [22] CAGACCAAGGAGGGAGAGTATGTGTGGGGACACTGG.

| Plasmid | **Method of cloning** | **Restriction Sites** | **Primers** |
| --- | --- | --- | --- |
| pEYFP-N1-DSC2a | Restriction cloning | *Xho*I, *Age*I | [1,2] |
| pEYFP-N1-DSC2a-p.N166Q | Site directed mutagenesis | *Xho*I, *Age*I | [3,4] |
| pEYFP-N1-DSC2a-p.T338V | Site directed mutagenesis | *Xho*I, *Age*I | [5,6] |
| pEYFP-N1-DSC2a-p.T340V | Site directed mutagenesis | *Xho*I, *Age*I | [7,8] |
| pEYFP-N1-DSC2a-p.N392Q | Site directed mutagenesis | *Xho*I, *Age*I | [9,10] |
| pEYFP-N1-DSC2a-p.N546Q | Site directed mutagenesis | *Xho*I, *Age*I | [11,12] |
| pEYFP-N1-DSC2a-p.T558V | Site directed mutagenesis | *Xho*I, *Age*I | [13,14] |
| pEYFP-N1-DSC2a-p.T560V | Site directed mutagenesis | *Xho*I, *Age*I | [15,16] |
| pEYFP-N1-DSC2a-p.N629Q | Site directed mutagenesis | *Xho*I, *Age*I | [17,18] |
| pEYFP-N1-DSC2a-p.T338V-p.T340V | Site directed mutagenesis | *Xho*I, *Age*I | [7,8,19,20] |
| pEYFP-N1-DSC2a-p.T558V-p.T560V | Site directed mutagenesis | *Xho*I, *Age*I | [15,16,21, 22] |
| pEYFP-N1-DSC2a-p.N166Q-p.N392Q | Site directed mutagenesis | *Xho*I, *Age*I | [3,4,9,10] |
| pEYFP-N1-DSC2a-p.N166Q-p.N546Q | Site directed mutagenesis | *Xho*I, *Age*I | [3,4,11,12] |
| pEYFP-N1-DSC2a-p.N166Q-p.N629Q | Site directed mutagenesis | *Xho*I, *Age*I | [3,4,13,14] |
| pEYFP-N1-DSC2a-p.T338V-p.T340V-p.T560V | Site directed mutagenesis | *Xho*I, *Age*I | [7,8,19,20,15,16] |
| pEYFP-N1-DSC2a-p.T338V-p.T340V-p.T558V-p.T560V | Site directed mutagenesis | *Xho*I, *Age*I | [7,8,19,20,15,16,13,14] |
| pEYFP-N1-DSC2a-p.N166Q-p.N392Q-p.N546Q | Site directed mutagenesis | *Xho*I, *Age*I | [3,4,9,10,11,12] |
| pEYFP-N1-DSC2a-p.N166Q-p.N392Q-p.N546Q-p.N629Q | Site directed mutagenesis | *Xho*I, *Age*I | [3,4,9,10,11,12,17,18] |
| pEYFP-N1-DSC2a-p.N166Q-p.T338V-p.T340V-p.T558V-p.T560V | Site directed mutagenesis | *Xho*I, *Age*I | [7,8,19,20,15,16,13,14,3,4] |
| pEYFP-N1-DSC2a-p.N166Q-p.T338V-p.T340V-p.N392Q-p.T558V-p.T560V | Site directed mutagenesis | *Xho*I, *Age*I | [7,8,19,20,15,16,13,14,3,4,9,10] |
| pEYFP-N1-DSC2a-p.N166Q-p.T338V-p.T340V-p.N392Q-p.N546Q-p.T558V-p.T560V | Site directed mutagenesis | *Xho*I, *Age*I | [7,8,19,20,15,16,13,14,3,4,9,10,11,12] |
| pEYFP-N1-DSC2a-p.N166Q-p.T338V-p.T340V-p.N392Q- p.N546Q-p.T558V-p.T560V-p.N629Q | Site directed mutagenesis | *Xho*I, *Age*I | [7,8,19,20,15,16,13,14,3,4,9,10,11,12,17,18] |
